# Selection of Reference Genes for Expression Study in Pulp and Seeds of *Theobroma grandiflorum* (Willd. ex Spreng.) Schum

**DOI:** 10.1371/journal.pone.0160646

**Published:** 2016-08-08

**Authors:** Lucas Ferraz dos Santos, Raner José Santana Silva, Daniel Oliveira Jordão do Amaral, Márcia Fabiana Barbosa de Paula, Loeni Ludke Falcão, Thierry Legavre, Rafael Moyses Alves, Lucilia Helena Marcellino, Fabienne Micheli

**Affiliations:** 1 Universidade Estadual de Santa Cruz (UESC), Departamento de Ciências Biológicas (DCB), Centro de Biotecnologia e Genética (CBG), Rodovia Ilhéus-Itabuna, km 16, 45662-900 Ilhéus-BA, Brazil; 2 Embrapa Recursos Genéticos e Biotecnologia, Brasília-DF, 70770-917, Brazil; 3 CIRAD, UMR AGAP, F-34398 Montpellier, France; 4 Embrapa Amazônia Oriental, 66095-903 Belém-PA, Brazil; Universidad Miguel Hernández de Elche, SPAIN

## Abstract

Cupuassu (*Theobroma grandiflorum* [Willd. ex Spreng.] Schum) is a species of high economic importance in Brazil with great potential at international level due to the multiple uses of both its seeds and pulp in the industry of sweets and cosmetics. For this reason, the cupuassu breeding program focused on the selection of genotypes with high pulp and seed quality—selection associated with the understanding of the mechanisms involved in fruit formation. Gene expression is one of the most used approaches related to such understanding. In this sense, quantitative real-time PCR (qPCR) is a powerful tool, since it rapidly and reliably quantifies gene expression levels across different experimental conditions. The analysis by qPCR and the correct interpretation of data depend on signal normalization using reference genes, i.e. genes presenting a uniform pattern of expression in the analyzed samples. Here, we selected and analyzed the expression of five genes from cupuassu (ACP, ACT, GAPDH, MDH, TUB) to be used as candidates for reference genes on pulp and seed of young, maturing and mature cupuassu fruits. The evaluation of the gene expression stability was obtained using the NormFinder, geNorm and BestKeeper programs. In general, our results indicated that the GAPDH and MDH genes constituted the best combination as reference genes to analyze the expression of cupuassu samples. To our knowledge, this is the first report of reference gene definition in cupuassu, and these results will support subsequent analysis related to gene expression studies in cupuassu plants subjected to different biotic or abiotic conditions as well as serve as a tool for diversity analysis based on pulp and seed quality.

## Introduction

Cupuassu (*Theobroma grandiflorum* [Willd. ex Spreng.] Schum) is a fruit species from the Amazon region that belongs to the Malvaceae family, as the cacao tree (*Theobroma cacao* L., used for chocolate fabrication) [1]. Cupuassu is a species of high economic importance in Brazil with great potential at international level due to the multiple uses of both its seeds and pulp. The pulp is used in the industry for candy, ice cream, liquor, and juice production [2, 3], while the seeds are used for the manufacturing of a product similar to chocolate called *cupulate*, as well as in the cosmetic industry [4, 5]. From the beginning of its domestication in the middle ‘70s, cupuassu plantations suffered production losses due to its susceptibility to the main pathogen of this species, the fungus *Moniliophthora perniciosa*, responsible for witches’ broom disease [6]. Because the Amazon region is the potential center of origin of both cupuassu and *M*. *perniciosa*, it is accepted that the fungus co-evolved with the plant and succeeded in surpassing the plant resistance through an adaptation process. For this reason, the cupuassu breeding program focused on the selection of genotypes with resistance to witches’ broom disease allied to high pulp and seed quality, both important characteristics to producers. Agronomic and molecular markers have been previously developed [7–9] and ESTs and SSR-ESTs have been identified [9], constituting the first step for subsequent deep molecular analysis of expressed sequences related to cupuassu resistance or quality. The expression analysis of sequences in which polymorphic markers are identified is an advantage, not only for the breeding program, but also for the understanding of the mechanisms involved in any of the physiological processes studied.

Quantitative PCR (qPCR) is one the most precise and sensitive techniques to analyze gene expression patterns in organisms subjected to biotic or abiotic stress, as well as throughout the developmental stages of such organisms [10]. The expression analysis of specific genes by qPCR depends on a signal normalization, which is achieved by using genes with a uniform pattern of expression in the considered samples, as referenced [11]. The normalization step is essential for a right and confident interpretation of the data obtained by qPCR [12, 13]. For this reason, it is necessary to previously test the stability of the expression of the candidate genes to be used as reference in the normalization, as carried out for other plant species [14–17] including cacao [18]. Such reference genes could be used in routine laboratory analysis by several research groups worldwide.

Here, we selected five genes from cupuassu EST databank [9] according to their putative functions, known in the literature as constitutive and which present good results as reference genes in other plant species. Such sequences were: i) tubulin (TUB) involved in cellular mechanism [19]; ii) glyceraldehyde-3-phosphate dehydrogenase (GAPDH), which participates in the Calvin cycle and glucose pathways [20, 21]; iii) actin (ACT), which is a cytoskeleton component and cell division regulator [22]; iv) malate dehydrogenase (MDH), an oxidoreductase from Krebs cycle involved in embryo metabolism and development and plant growth [23–25]; and v) acyl carrier protein (ACP) involved in fatty acid biosynthesis [26]. With the advance of the qPCR technique, some software packages supporting reference gene stability analysis also were developed. Among them, NormFinder, geNorm and BestKeeper programs allow the statistical identification of the best internal controls inside a group of candidate genes for normalization [27–29]. The use of such statistical algorithms simplifies the selection of adequate reference genes by calculation of the stability. Here, we analyzed by qPCR the expression pattern of the five candidate genes described above, potentially useful as reference genes for expression studies in cupuassu.

## Material and Methods

### Primer design for qPCR

Five gene sequences previously obtained from cupuassu [9] were used: tubulin (TUB), glyceraldehyde 3-phosphate dehydrogenase (GAPDH), actin (ACT), malate dehydrogenase (MDH), and acyl carrier protein (ACP). The sequences are available in the S1 Table. Sequences were aligned with the cacao genome using the Blast tool available in the database CocoaGenDB (http://cocoagendb.cirad.fr/gbrowse/tools.html). Specific primers were designed using the Primer3Plus program (http://www.bioinformatics.nl/cgi-bin/primer3plus/primer3plus.cgi), and the following parameters were analyzed: amplicon size 100–250 pb, GC content of 40–60%, primer size of 20–23 bp and annealing temperature of 60°C. To avoid cross-reaction between genes, the amplified regions were checked for the following characteristics: size difference, melting temperature, GC content, and GC/AT ratio [30] (S2 Table)

### Plant material

Samples were collected from the cupuassu genotype 174 (Coari) maintained in the field at Embrapa Amazônia Oriental (PA, Brazil) and selected in the frame of the breeding program of this institution for its characteristics of resistance to witches’ broom disease and fruit quality. Seed and pulp were collected from young, maturing, and mature fruits. For each developmental stage and for each tissue, three biological samples were collected (1 biological unit = 1 fruit); a total of nine seed samples (3 units x 3 developmental stages) and nine pulp samples (3 units x 3 developmental stages) were obtained.

### RNA extraction and cDNA synthesis

Total RNA was extracted from the samples described above using the CTAB method [31, 32] with modifications [33]. Cupuassu tissues were ground in liquid nitrogen and the extraction carried out with buffer containing 2% CTAB (w/v), 2 M NaCl, 100 mM Tris, pH 8.0, 25 mM EDTA, pH 8.0, 2% PVP 10,000 (w/v), and 2% β-mercaptoethanol (v/v). Samples were then treated with chloroform, and the total RNA was precipitated with 2 M of LiCl overnight at 4°C. RNA was ressuspented in SSTE buffer (1 M NaCl, 0,5% SDS, 10 mM Tris-HCl pH8, 1 mM EDTA pH8), then extracted with 1 volume of phenol, followed by two extractions with chloroform/isoamyl alcohol (24:1), and then precipitated with 2.5 volumes of ethanol 100%. After resuspension in DEPC water, the RNA was quantified using the Nanodrop 2000 spectrophotometer (Thermo Scientific). Afterwards, 1 μg of RNA was treated with DNase I according to the manufacturer’s recommendations (Fermentas Lifes Sciences). The integrity of the RNA was checked on 1% agarose electrophoresis gel. The cDNA synthesis was performed in a final volume of 20 μl using the cDNA RevertAid First Strand cDNA Synthesis Kit according to the manufacturer’s recommendations (Thermo Scientific). The cDNA were quantified by Nanodrop 2000 (Thermo Scientific), and 100 ng/μl of each cDNA sample were used for the qPCR analysis.

### Quantitative PCR

Expression analysis by qPCR was performed using standard settings of the Stratagene MX3005P system (Agilent Technologies). The qPCR reaction consisted of 100 ng/μl of cDNA, 0.3 μM of each primer from candidate reference genes (Table 1), and 1X of Maxima^™^ SYBR Green/ROX qPCR Master Mix (Thermo Scientific) in a total volume of 12.5 μl. Cycling conditions were: 50°C for 2 min., 95°C for 10 min followed by 40 cycles at 95°C for 15s, 58°C for 35s, and 72°C for 30s, with detection of the fluorescent signal at the end of each extension cycle. To verify that each primer pair produced only a single PCR product, a dissociation analysis was carried out under the following cycling conditions: 95°C for 1 min, 55°C for 30 s, and 95°C for 30 s, and it was analyzed with MxPro QPCR software (Agilent Technologies). The gene expression level was analyzed on 3 experimental replicates for all samples. Experiments included a negative control (no template DNA), and amplification efficiency of each primer pair was analyzed using two dilutions [1:50 and 1:100 (v/v)] of each cDNA sample. Real-time data acquisition was performed by the Stratagene MX3005P system containing the MxPro QPCR software (Agilent Technologies), which provided the values of cycle threshold (Ct) and of fluorescence. Amplification efficiency (E) was accessed using Miner 2.2 software [34]

**Table 1:** Candidate reference genes, primer characteristics, and efficiency obtained by RT-qPCR. CV: coefficient of variation, F: forward; R: reverse.

Gene	Cell function	Primers (5′-3′)	Amplicon size (bp)	Efficiency	R^2^	CV
ACP	Fatty acid and polyketide biosynthesis	F: AGGATGCAGCTGACCTGATT R: AACTTGGGGTGGATTCATCA	177	0.86	88	5.5
ACT	Cytoskeleton structure protein	F: TGAGTTCACTTGACACAGGACA R: CCTTCCAGCAGATGTGGATT	153	0.88	98	8.54
GAPDH	Glycolysis and gluconeogenesis	F: TGACTTGATCCGACACATGG R: TCCCATCCATCCAAAAACAT	154	0.88	90	4.68
MDH	Citric acid cycle and gluconeogenesis	F: ATTCAGAAGGGCGTTTCCTT R: CCATAGCACAGGGTTTTGGT	164	0.86	95	3.43
TUB	Cytoskeleton structure protein	F: ATGTTGGTGAGGGAATGGAG R: TTCAGCCACCAAATCTGTGA	215	0.86	91	6.85

### Reference gene stability analyses

To establish the ideal reference gene among the diverse biological conditions, gene expression data were analyzed using the statistical approach used by the geNorm, NormFinder and BestKeeper software. The geNorm VBA applet for Microsoft Excel determines the most stable reference genes from a set of tested genes in a given cDNA sample panel, and it calculates a gene expression normalization factor (NF) for each tissue sample based on the geometric mean of a user-defined number of reference genes [28]. The geNorm program calculates the gene expression stability measure *M* for a reference gene as the average pairwise variation *V* for that gene with all other tested reference genes. Stepwise exclusion of the gene with the highest *M* value allows for ranking of the tested genes according to their expression stability [28]. The geNorm program also determines the optimal number of reference genes needed for qPCR experiment using pairwise variation (V_n_/V_n+1_) analysis between the normalization factors (NF_n_ and NF_n+1_). The NormFinder program uses Ct values transformed into relative quantities (Q), estimated by Q = *E* (Ct minimum—Ct sample), where *E* is the amplification efficiency and the Ct minimum is the lowest Ct value (sample with the highest expression among all samples) [28]. The NormFinder program identifies the best reference gene by ranking all candidates to the reference genes, according to their expression stability in a given sample set and experimental conditions. NormFinder calculates not only the overall variation of candidate normalization genes, but also the variation between subgroups from the sample set under investigation. The BestKeeper program determines the best reference genes and combines them into an index, which can be used as standard in the same way such as any single reference gene [29]. The BestKeeper Excel template offers three main measures to detect the most stable genes: the standard deviation (SD) of the Ct of all samples for one gene, the correlation coefficient with the BestKeeper index (*r*), and the coefficient of variation (CV) of a potential reference gene [29, 35]. The BestKeeper guideline does not indicate which of these three measures is more reliable nor has more weight to select the best reference gene. According to previous studies, only SD, only *r*, or a combination of SD+CV have been already used [36–38]. On the other hand, each potential reference gene was evaluated for the three measures offered by the BestKeeper (SD, *r*, and CV), and the mean of the rankings was calculated to determine the final rank of each gene, as described by Olias and collaborators [35].

## Results and Discussion

### Candidate reference gene selection, primer design and efficiency analysis

Based on previous studies, the ACP, ACT, GAPDH, MDH, and TUB genes were selected as possible candidate reference genes in cupuassu expression analysis. All these genes presented constitutive expression patterns in other plant species and some of them, such as ACP, presented a special interest for the *Theobroma* genus. The ACP gene is an important co-factor of several metabolic pathways [39], including those related to lipid storage occurring in seeds, and for this reason it was previously chosen in cacao [18] and also may be a good candidate for cupuassu. The MDH gene is also an interesting candidate due to its involvement in plant growth and embryo development [25], one subject addressed here (fruit under maturation process). Specific primers for each candidate’s reference genes (Table 1) were tested in all samples (i.e. pulp and seed, each one in the 3 maturation fruit stage—see Material and methods) to confirm the fragment size and the amplification of a single PCR product. Dissociation curves showed that after 35 cycles of amplification, all the primers used for the analysis presented a unique peak (i.e., a unique PCR product) proving the primer specificity (S1 Fig). Both cDNA dilution 1:50 and 1:100 were tested, and no significant difference was observed (data not shown); thus, the 1:100 dilution was used.

### Expression profile of candidate reference genes

**Fig 1:**
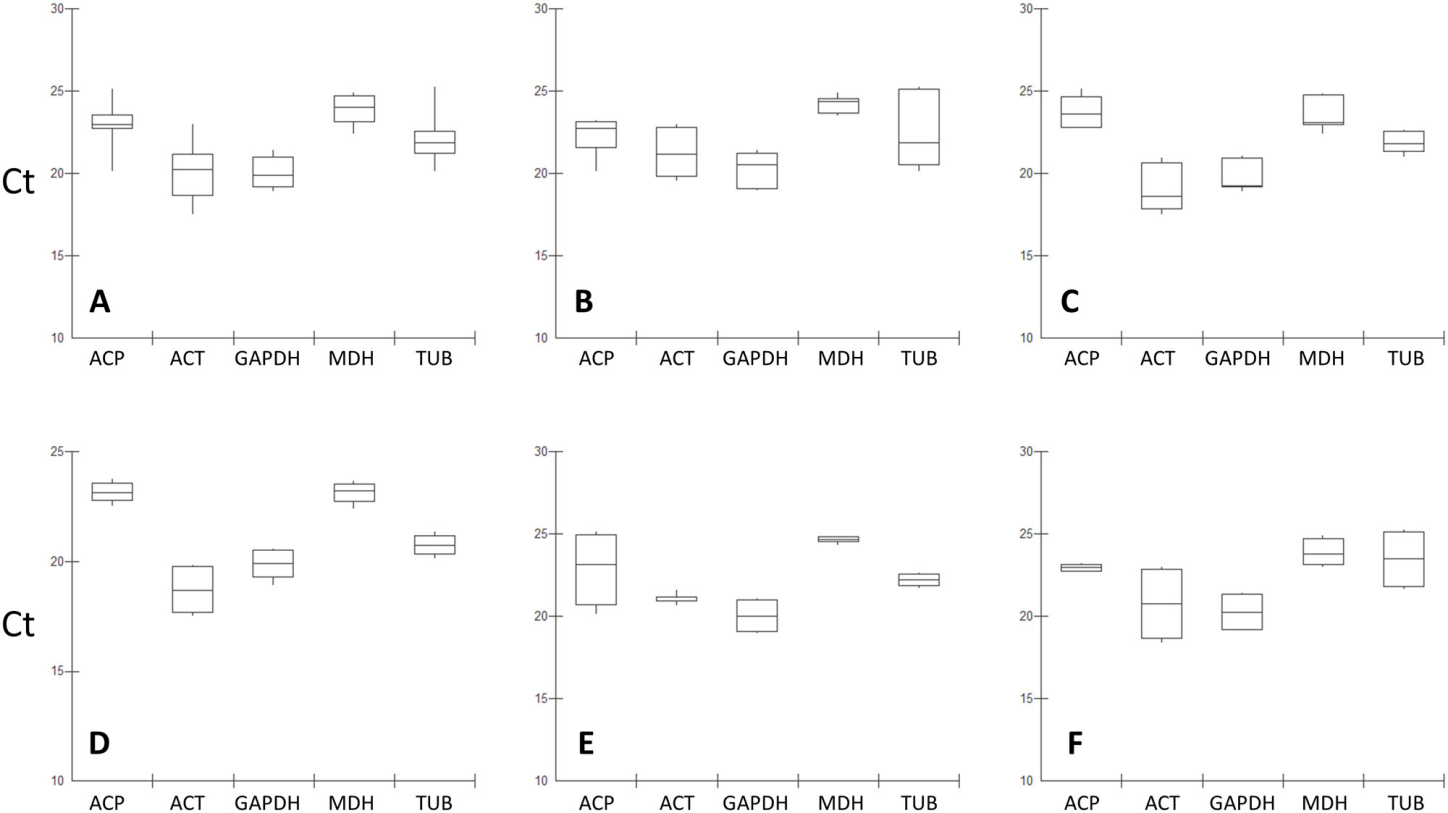
Box-plots showing Ct variation for each candidate reference gene estimated by RT-qPCR for the different samples. **A.** All samples (all tissues, all developmental stages). **B.** Pulp samples (all developmental stages). **C.** Seed samples (all developmental stages). **D.** Young fruit samples (all tissues). **E.** Maturing samples (all tissues). **F.** Mature samples (all stages). The line inside the box represents the median; the two parts of the box on each side of the line indicated the 25 and 75 percentiles. Whiskers represent the maximum and minimum values.

The raw gene expression levels were estimated by Ct for all the samples—all together or according to the tissue (pulp *vs* seeds) or to the developmental stage (young, maturing or mature fruit) (Fig 1). Mean Ct values presented variations for each gene between samples: Ct varied from 22.2 to 24.0 for ACP, from 18.7 to 21.3 for ACT, from 19.8 to 21.3 for GAPDH, from 23.1 to 24.9 for MDH, 20.7 to 23.5 for TUB (S3 Table; Fig 1A to 1F). Generally, the ACT and GAPDH genes presented the lowest Ct using the different cupuassu samples (Fig 1A, 1B, 1C, 1D and 1F), while ACP presented the highest (Fig 1A, 1C, 1D and 1E). The global Ct variation was low—from 18.7 (for ACT) to 24.9 (for MDH)–and was consistent (similar or lower) with the one observed in other studies of references genes previously published in other organisms [12, 16, 35, 40]. Such low Ct variation also indicated that the candidate genes presented similar behavior regardless of the biological condition analyzed (Fig 1; S3 Table) and for this reason may be used in combination (2 or 3 reference genes) for RT-qPCR normalization.

### Expression stability analysis

**Fig 2:**
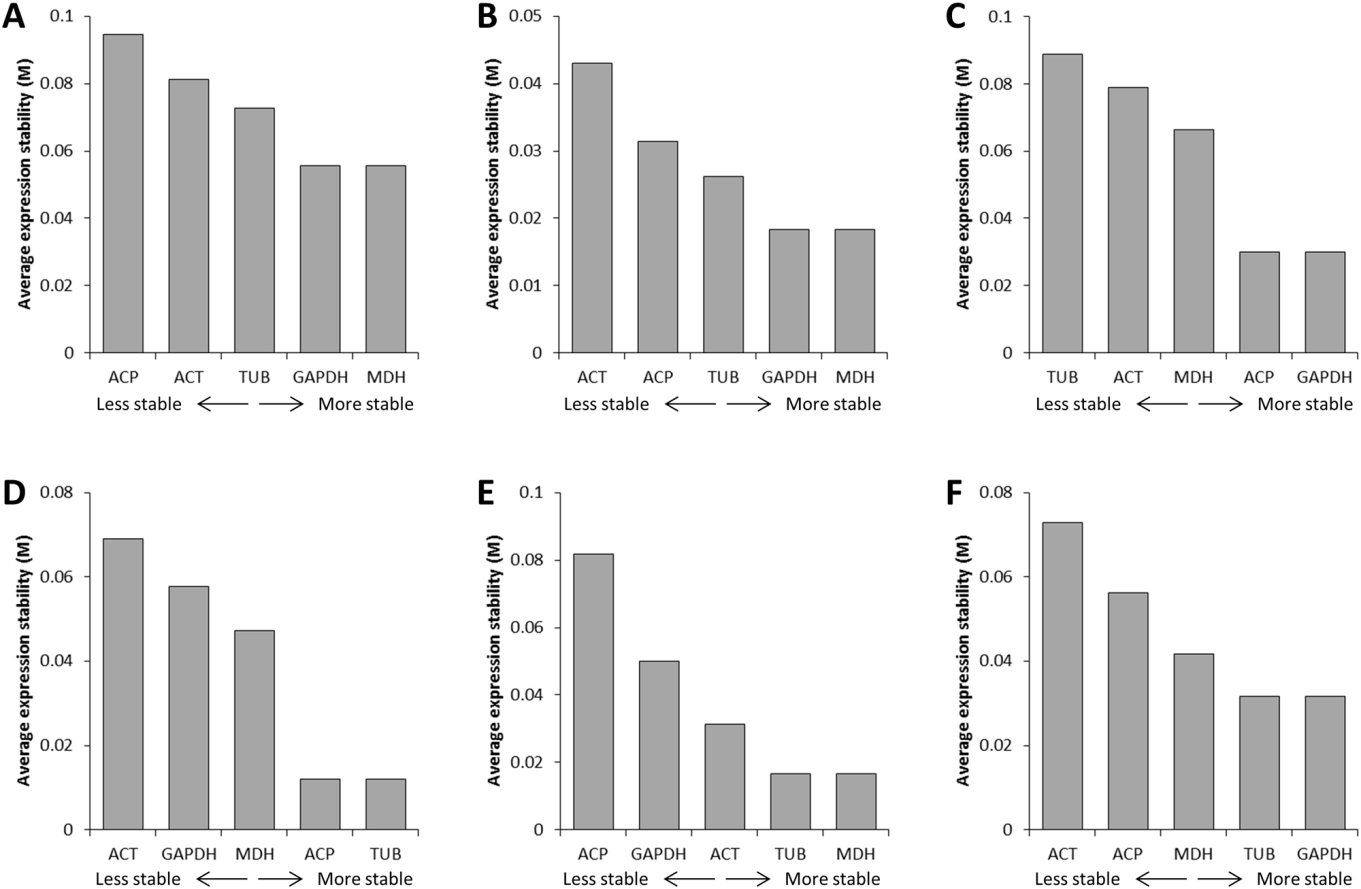
Gene expression stability obtained by geNorm. **A.** All samples (all tissues, all developmental stages). **B.** Pulp samples (all developmental stages). **C.** Seed samples (all developmental stages). **D.** Young fruit samples (all tissues). **E.** Maturing samples (all tissues). **F.** Mature samples (all stages). Mean expression stability (*M*) was calculated following stepwise exclusion of the least stable gene across all the treatment groups.

The expression stability of the five reference candidate genes was analyzed using the geNorm, NormFinder, and BestKeeper programs. The stability was evaluated in cupuassu pulp and seeds during the fruit development stages: i) all stages and tissues put together; ii) according to the tissue; and iii) according to the fruit developmental stage. In our conditions, the geNorm program estimated that the optimal number of reference genes was at least 3 according to the samples analyzed (cut-off of 0.15; S4 Table). However, as the guideline, the authors of the geNorm program indicated that the proposed 0.15 value must not be taken as a too strict cut-off for determination of the optimal number of reference genes. It can be that a valid normalization strategy is obtained just using the two best reference genes, resulting in a much more accurate and reliable normalization than if one single reference gene is used [41]. The geNorm program organized the candidate genes according to their stability (Fig 2). In the all stages/tissues sample and in the pulp sample, the two more stable genes were GAPDH and MDH (Fig 2A and 2B). In seeds, ACP and GAPDH were the most stable genes (Fig 2C). In fruit samples, ACP and TUB (young fruit), TUB and MDH (maturing fruit), and TUB and GAPDH (mature fruits) were the most stable, respectively (Fig 2D to 2F).

The NormFinder program uses an algorithm for identification and optimization of reference genes among a candidate group. It ranked the candidate reference genes according to their stability of expression for a sample group (e.g., pulp) and according to the experimental design (e.g., throughout the fruit development). The algorithm is based on a mathematic model of gene expression and used a strong statistic network to estimate not only the global variation of expression of the candidate reference genes, but also the variation between the sample sub-groups from the initial sample set [27]. In the all stages/tissues samples, GAPDH and MDH were the most stable genes (0.0189 and 0.0182, respectively), as well as in the young fruit sample (0.011 for both; Table 2). In the pulp, MDH and TUB presented the best expression stability (0.0047 and 0.0091, respectively; Table 2), as well as for the maturing fruit sample (0.007 for both; Table 2). In seeds, ACT and TUB were the most stable genes (0.0197 for both), while in the mature fruit, GAPDH and TUB presented the best stability (0.013 and 0.014, respectively; Table 2). As a general analysis made by the NormFinder program, the best combination of two genes for normalization in cupuassu samples was GAPDH and MDH (0.014; Table 2). Generally, the result we obtained here presented very high quality and stability compared with other works; our best combination presented a stability of 0.014, much lower than the one selected in other studies (0.06) [42]–value that corresponds with our worse stability (ACP; Table 2).

**Table 2:** Expression stability of the candidate reference genes obtained with NormFinder program. Data in bold indicates the best two genes for the sample group analyzed.

	ACP	ACT	GAPDH	MDH	TUB	Best combination of two genes*
All stages/all tissues	0.061	0.0595	**0.0189**	**0.0182**	0.0342	GAPDH and MDH (0.014)
Pulp (all stages)	0.0862	0.0185	0.0347	**0.0047**	**0.0091**	
Seeds (all stages)	0.1190	**0.0197**	0.1013	0.0772	**0.0197**	
Young fruit (all tissues)	0.118	0.015	**0.011**	**0.011**	0.122	
Maturing fruit (all tissues)	0.19	0.011	0.08	**0.007**	**0.007**	
Mature fruit (all tissues)	0.14	0.022	**0.013**	0.138	**0.014**	

* Given by the NormFinder program for the overall analysis. The best combination value is indicated inside brackets.

The BestKeeper program allowed the determination of three main measures (SD, *r*, and CV; see Material and methods) which when combined resulted in a gene ranking (Table 3). The ranking indicated that GAPDH and MDH were the best genes in the all stages/all tissues sample, GAPDH and TUB in the pulp sample, GAPDH and MDH in seeds, MDH and GAPDH/TUB in the case of young fruit, MDH and TUB for maturing fruit, and ACP and GAPDH/MDH for mature fruits (Table 3). Even if the calculation and statistics used in the geNorm, NormFinder and BestKeeper programs were different, the results obtained for the normalization of the expression in cupuassu tissues were slightly the same. As presented in Table 4, the GAPDH and MDH genes were present both or individually in all the combinations of two or three best reference genes. Generally, the third-best gene was TUB, followed by ACT or ACP (Table 4).

**Table 3:** Best reference genes obtained by the BestKeeper program. SD: standard deviation; *r*: coefficient of correlation; CV: coefficient of variation. Best reference genes are indicated in bold.

		ACP	ACT	GAPDH	MDH	TUB
All stages/all tissues	SD*	0.92 (3)	1.49 (5)	0.91 (2)	0.77 (1)	1.16 (4)
	CV*	4.02 (2)	7.40 (5)	4.53 (3)	3.24 (1)	5.22 (4)
	r*	0.383 (5)	0.854 (3)	0.871 (1)	0.831 (4)	0.855 (2)
	Mean of rankings	3.33	4.33	**2**	**2**	3.33
Pulp (all stages)	SD*	0.79 (2)	1.21 (5)	0.82 (3)	0.85 (4)	0.49 (1)
	CV*	3.33 (2)	6.36 (5)	4.12 (4)	3.63 (3)	2.25 (1)
	r*	0.871 (5)	0.982 (3)	0.988 (1)	0.987 (2)	0.944 (4)
	Mean of rankings	3	4.33	**2.67**	3	**2**
Seeds (all stages)	SD*	0.98 (3)	1.11 (4)	0.83 (2)	0.45 (1)	1.82 (5)
	CV*	4.44 (3)	5.23 (4)	4.11 (2)	1.87 (1)	8.12 (5)
	r*	0.609 (5)	0.877 (2)	0.751 (3)	0.659 (4)	0.959 (1)
	Mean of rankings	3.66	3.33	**2.33**	**2**	3.66
Young fruit (all tissues)	SD*	0.45 (3)	1.04 (5)	0.68 (4)	0.44 (2)	0.44 (2)
	CV*	1.94 (2)	5.55 (5)	3.43 (4)	1.89 (1)	2.15 (3)
	r*	-0.941 (5)	0.969 (2)	0.991 (1)	0.936 (3)	-0.910 (4)
	Mean of rankings	3.33	4	3**	**2**	3**
Maturing fruit (all tissues)	SD*	2.13 (5)	0.22 (2)	0.98 (4)	0.17 (1)	0.38 (3)
	CV*	9.31 (5)	1.04 (2)	4.91 (4)	0.70 (1)	1.73 (3)
	r*	0.995 (1)	-0.738 (5)	0.992 (2)	0.915 (4)	0.984 (3)
	Mean of rankings	3.66	3	3.33	**2**	**3**
Mature fruit (all tissues)	SD*	0.19 (1)	2.13 (5)	1.06 (3)	0.81 (2)	1.69 (4)
	CV*	0.84 (1)	10.30 (5)	5.23 (3)	3.38 (2)	7.20 (4)
	r*	0.986 (4)	1.000 (1)	0.998 (2)	0.987 (3)	1.000 (1)
	Mean of rankings	**2**	3.66	2.66**	2.66**	3

* Rank is indicated inside brackets.

** Same ranking for a given condition.

**Table 4:** Main reference genes indicated for each physiological condition using the geNorm, NormFinder and BestKeeper programs. Genes are indicated in increasing order of stability.

	geNorm	NormFinder	BestKeeper
All stages/all tissues	MDH/GAPDH/TUB	MDH/GAPDH/TUB	GAPDH/MDH
Pulp (all stages)	MDH/GAPDH/TUB	MDH/TUB/ACT	GAPDH/TUB
Seeds (all stages)	GAPDH/ACP/MDH	ACT/TUB/MDH	GAPDH/MDH
Pulp and seeds (young fruit)	ACP/TUB/MDH	MDH/GAPDH/ACT	MDH/ GAPDH or TUB
Maturing pulp and seeds	MDH/TUB/ACT	MDH/TUB/ACT	MDH/TUB
Mature pulp and seeds	GAPDH/TUB/MDH	GAPDH/TUB/ACT	ACP/GAPDH or MDH

A reference gene needs to present constant expression level, but the stability of this gene may suffer variations according to the tissue or biological condition analyzed such as developmental stage or response to stress [43, 44]. All genes evaluated presented stability values adequate for the normalization of the target gene in expression studies using RT-qPCR, with a highlight for the GAPDH and MDH (Table 4). According to three different gene-expression stability analysis programs, these two genes presented high stability in all the tested conditions and also presented compatibility to be used together in RT-qPCR expression analyses (Table 2). These results are consistent with studies carried out for other plant species such as in coffee seeds, *Vitis vinifera* fruit pericarp, developing organs/tissues, or plants under stress, where GAPDH has been identified as one of the most stable among the tested genes [18, 44–46]. MDH was also considered as a good reference gene in similar analysis in Pearl millet [47] and in *Theobroma cacao* [18].

## Conclusion

In general, our results indicate that the GAPDH and MDH genes constituted the best combination as reference genes to analyze gene expression in cupuassu samples. In the third position, the TUB gene may be envisaged. To our knowledge, this is the first report of reference gene identification in cupuassu. This analysis will support subsequent expression studies in cupuassu plants subjected to different biotic or abiotic conditions as well as serve as a tool for diversity analysis based on pulp and seed quality.

## Supporting Information

S1 Fig Dissociation curves obtained for the five genes used in this study. Temperature is presented in the x-axis while the fluorescence is presented in the y-axis.
(DOCX)

S1 Table Five sequences of cupuassu used in this study. (DOCX)

S2 Table Amplicon characteristics. (DOCX)

S3 Table Ct mean for each reference candidate gene in the different tissues and stages analyzed. Maximum and minimum are indicated in bold. (*) indicates two maximum values for the considered gene.
(DOCX)

S4 Table Estimation of the optimal number of reference genes required for accurate normalization based on pairwise variation (Vn/n+1) analysis. The asterisk indicates the lowest V value in each condition. Bold indicate the variation ≤ 0.15 (suggested cut-off).
(DOCX)

## Abbreviations

EST: expressed sequence tags
SSR: simple sequence repeat
qPCR: quantitative PCR
TUB: tubulin
GAPDH: glyceraldehyde-3-phosphate dehydrogenase
ACT: actin
MDH: malate dehydrogenase
ACP: acyl carrier protein
NF: normalization factor
SD: standard deviation
CV: coefficient of variation
