# Supplementary material for: Selection of Reference Genes for Expression Study in Pulp and Seeds of Theobroma grandiflorum (Willd. ex Spreng.) Schum
Source: PLoS One. 2016 Aug 8;11(8):e0160646. doi: 10.1371/journal.pone.0160646 (PMC4976894; doi:10.1371/journal.pone.0160646)
Supplement: S1 Fig — Temperature is presented in the x-axis while the fluorescence is presented in the y-axis. (DOCX) [file pone.0160646.s001.docx]

**S1 Fig.** Dissociation curves obtained for the five genes used in this study. Temperature is presented in the x-axis while the fluorescence is presented in the y-axis.

**
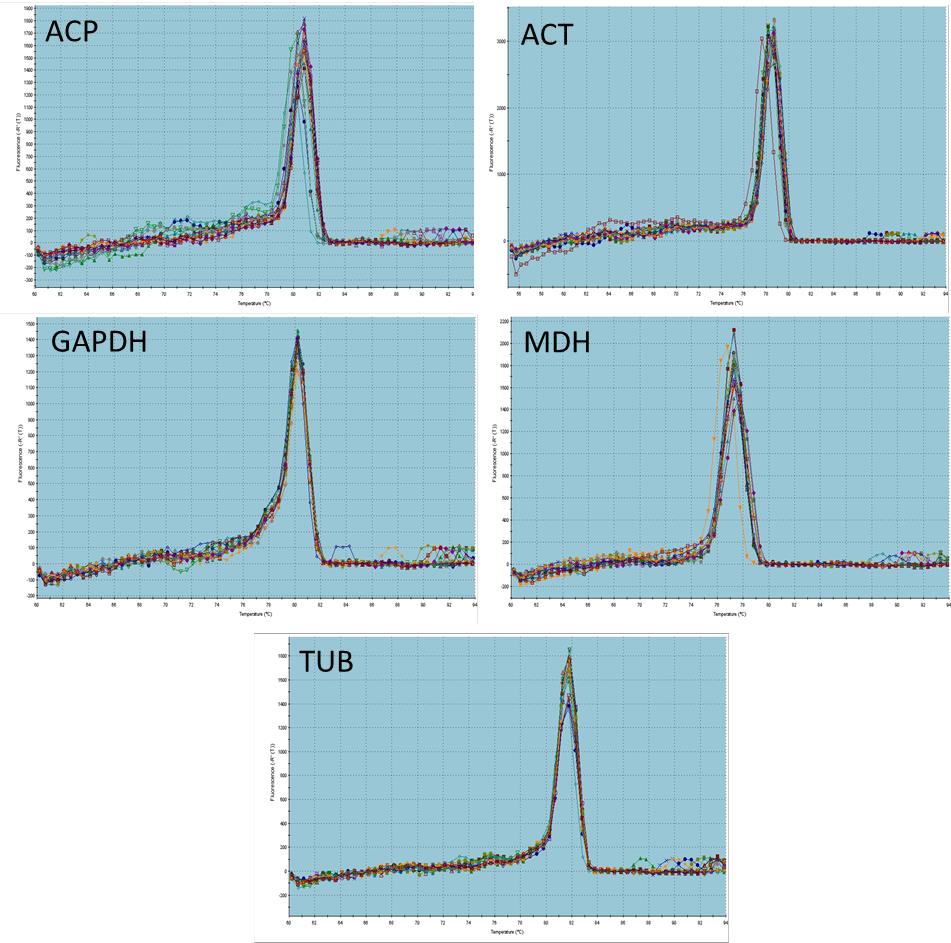
**
