# Supplementary material for: Selection of Reference Genes for Expression Study in Pulp and Seeds of Theobroma grandiflorum (Willd. ex Spreng.) Schum
Source: PLoS One. 2016 Aug 8;11(8):e0160646. doi: 10.1371/journal.pone.0160646 (PMC4976894; doi:10.1371/journal.pone.0160646)
Supplement: S1 Table — (DOCX) [file pone.0160646.s002.docx]

**S1 Table.** Five sequences of cupuassu used in this study. Primers used for qPCR are underlined.

| >**TgACP-c2265** – 633 bp  AAACCAGAGACAGTGAACAAAGTTTGTGAAATAGTGAGGAAGCAACTGGCACTACCTGATGATTCTCCATCACCGGGGAGTCAAAGTTTCTACCTTGGAGCTGATTCCCTTGACACGGTCGAGATTGTGATGGGACTCGAGGAGGAGTTCGGAATCATTGTGGAAGAAGAAAGTGCCCAGAGTATTAGCACTGTTCAGGATGCAGCTGACCTGATTGAGAAGCTCATGGATAAGAACTGTGCTTAAATTGCAAACAGCCCAACACAAGTCACTTATGAGCCCCTCCTATGTTGGCATGCCGTCGGCTTTGTTTTGTTGCTTATGGAATGTTTAAAACCTAATAGTATCTTACCTGATGAATCCACCCCAAGTTTGGCTGTTCACCTTTATCCTTACAGCTTATGTAGAACTCCAGTGTCTCATTTCCCTGTTAAGCTTAGGCATTTGTTTCATTATGTTATCATAAAATACATTGCTCAAGGCCATAGAGCATGTTTCCACCCGGGAAATCTATAAATTATGCTGCTTACTACTTTACTTGCATTGCTCACTTTTCACAGAGGGAAATGGGGTTGGGCTTAGGATGATAACCCAAGAAAACCCGACTAGACCCGAAATCTGTGAGCAGACCAC |
| --- |
| >**TgTUB-c547** – 1511 bp  GGAACTTTACTGTCTCGAGCATGGCATTCAGCCTGATGGCCAAATGCCAAGTGATAAGACTGTTGGTGGAGGAGACGATGCTTTCAACACCTTTTTCAGCGAAACTGGTGCCGGAAAGCATGTCCCTCGTGCCGTATTTGTTGATCTCGAGCCTACTGTCATCGATGAAGTGAGGACTGGAACGTACCGCCAGCTGTTCCACCCTGAGCAACTAATCAGTGGCAAAGAAGATGCTGCCAACAATTTTGCTCGTGGCCATTACACAATTGGAAAAGAGATTGTCGATCTCTGCTTGGATCGTATCCGAAAGCTTGCGGATAACTGTACTGGGCTACAAGGATTCTTGGTCTTTAATGCAGTTGGAGGTGGTACTGGTTCTGGTCTTGGATCCCTTCTCTTGGAGCGTCTCTCTGTTGACTACGGAAAGAAGTCCAAGCTTGGTTTCACTGTCTATCCTTCACCTCAGGTTTCTACATCTGTTGTAGAGCCTTACAACAGTGTGCTGTCCACCCATTCGCTCCTTGAGCACACTGATGTTGCTGTGCTTCTTGATAACGAAGCAATTTATGACATCTGCAGGCGATCTCTGGACATTGAACGGCCCACTTACACCAATCTTAACCGCCTTGTCTCTCAGGTTATCTCATCTCTTACTGCCTCTTTGAGGTTTGATGGGGCCTTGAATGTGGATGTGACTGAGTTCCAGACTAACCTGGTCCCCTACCCCAGGATCCATTTTATGCTTTCCTCATATGCCCCTGTTATTTCTGCTGAGAAGGCTTACCATGAGCAATTGTCAGTGGCTGAGATCACCAACAGTGCATTTGAGCCTTCTTCCATGATGGCCAAATGTGACCCACGCCATGGGAAGTACATGGCCTGCTGCCTCATGTACCGAGGTGATGTTGTGCCCAAAGATGTGAATGCTGCTGTGGCCACTATCAAGACCAAGCGAACTATCCAATTTGTCGATTGGTGCCCAACTGGATTTTAAGTGTGGTATCAACTACCAGCCACCAACTGTGTTCCAGGAGGTGACCTTGCCAAGGTGCAGAGGGCCGTCTGCATGATCTCTAACTCAACCAGTGTTGCAGAAGTGTTCTCCCGCATCGACCACAAATTTGATCTCATGTATGCCAAGCGTGCCTTTGTGCACTGGTATGTTGGTGAGGGAATGGAGGAAGGAGAGTTCTCAGAGGCTCGTGAGGATCTTGCTGCACTGGAAAAAGATTATGAGGAAGTTGGTGCTGAGTCTGGTGAAGGAGATGAAGGGGATGAGGGCGAGGAGTACTGAGGGAAGGACTGCCTTCATATATTTGCTACTCAGATGTTCTTCGTGTTGCTTCCAATTTATGTCACAGATTTGGTGGCTGAATCTCTTTTTTTGAATGTTTTTGGTTACTTGGGGTGACGTATCCAGTTCGTCATATTTTCAATGAATAAGTTTTAGTAAAGACTTTGCCGTATGGGAGGTTTCCAAGCCTTTAGGACCATTGTAATGGAAGGTCTAA |
| >**TgGAPDH-c136** - 1278 bp  TTTTGGTCGTCTTTGTTAGAGATTGTTTCTCGATTTTCGGACATGGCGAAGATCAAGATCGGAATCAACGGATTTGGAAGAATCGGGCGTTTGGTTGCCAGAGTCGCTCTCCAAAGCGATGATATTGAGCTTGTTGCTGTTAACGATCCTTTTATCAGCGTTGAATACATGATCTACATGTTTAAGTACGATAGTGTTCACGGTCAATGGAAGCACCATGAGCTTAAGGTGAAGGACTCAAAGACCCTTCTTTTTGGTGAAAAGCCTGTCACTGTTTTCGGCATCAGAAACCCTGAGGAAATCCCCTGGGGGGAGACTAGGAGCTGAATATGTTGTTGAGTCTACTGGTGTTTTCACCGACAAGGACAAAGCTGCTGCTCACTTGAAGGGTGGCGCAAAGAAGGTGATCATTTCTGCTCCCAGTAAGGATGCCCCCATGTTTGTAATGGGTGTCAATGAGAAGGAATACAAGCCTAATCTTGATATTGTCTCCAATGCTAGCTGCACTACCAACTGCCTTGCTCCTTTGGCTAAGGTCATCAATGACAAATTTGGCATTGTTGAGGGTCTTATGACCACTGTCCATTCAATTACTGCTACCCAAAAGACTGTTGATGGTCCCTCAATGAAGGACTGGAGAGGTGGTAGAGCTGCTTCCTTCAACATCATTCCCAGCAGCACTGGAGCTGCCAAGGCTGTTGGCAAGGTGTTACCTGCGTTGAATGGCAAGCTGACTGGAATGGCTTTCCGTGTTCCCACTGTTGATGTCTCTGTGGTTGACCTCACTGTGAGACTTGAGAAGCCAGCTTCTTATGAGGATATTAAGAAGGCTGTCAAGGAGGCATCTGAAACCCACATGAAGGGAATTCTTGGTTATGTAGATGAAGATTTGGTGTCATCTGACTTTGTTGGTGACCAAAGGTCAAGCATTTTTGATGCCAAGGCTGGAATTGCTTTGAATGATAAGTTTGCTAAGCTTGTTACATGGTATGACAACGAGTGGGGTTACAGTTCCCGTGTTGTTGACTTGATCCGACACATGGCGTCTTGCAAGTGAGTGATATTGGTGTAGGAATGCGTGTTGTGGGCCTCCTCTTTCATCAGCCCTAGCAATTTGGGAGAGGAGATGTCATGGGAATAATAGATTTGTACGGGACATGTTTTTGGATGGATGGGAGTGTTTCCATTTTTATGGAGTATTTGCATTTTTGGCTAGAACAGTTTTCATTCTGTCGGAACGAGTTTACTATTTTGGTTTGTTCATAATTTCACTAACTC |
| >**TgACT-c3987** – 456 bp  AGACTTTGAGTTCACTTGACACAGGACACAAAAAAGCCAACTAAATGGAAAATGTAACTCACCATCAAAGCACTAATAAAACTTAGAAGCACTTCCTGTGGACAATGGATGGACCAGATTCATCATACTCGCCCTTGGAAATCCACATCTGCTGGAAGGTGCTGAGCGATGCCAAGATAGATCCTCCAATCCAGACACTGTACTTTCTCTCTGGTGGTGCAACGACCTTAATCTTCATGCTGCTTGGAGCAAGGGCAGTGATCTCCTTGCTCATTCGGTCTGCAATACCAGGGAACATAGTTGAACCACCACTGAGCACAATGTTACCATAGAGATCTTTCCTGATATCCACATCACACTTCATGATAGAGTTGTAGGTAGTCTCATGGATTCCAGCAGCTTCCATCCCAATAAGAGATGGCGTGGAAGAGGACTTCTGGACAACGGAACTCTCTC |
| >**TgMDH-c1193** – 543 bp  GGCGGTACGGAGGTTGTTGAGGCAAGGCTGGTGCTGGGTCTGCAACATTATCAATGGCATATGCTGCGGTTAAATTTGCTGATGCATGCCTTCGTGGCTTAAGAGGAGATGCTGGTGTTGTTGAATGTGCATTTGTGGCCTCACATGTGACTGAATTACCCTTCTTCGCATCCAAGGTAAGACTTGGTCGGTTCGGAGTTGAGGAAGTATACCCTCTTGGCCCACTAAATGAGTATGAGAGGGTTGGCTTGGAGAAGGCAAAGAAAGAACTAGCAGGTAGCATTCAGAAGGGCGTTTCCTTTGTCAAGAAATGAATAGGTGCTCATATCTTGGTTTCCCCTGAGAATCAAGGAACACTACAGTTATACCTATTTTCTGATAACAAAAGTTTCTAGGAATAAGGGGTAAAACGCCTGTTTTGTTTTACCAAAACCCTGTGCTATGGGTTCCTGGATGTGAGCCAACCTCGCAAATGTGGTGGGTCATGGCTAAAATTCATAATACATTAATGGTTTGTAAATTAGGAACGTCTGTTGCATTTTT |
