# Supplementary material for: Selection of Reference Genes for Expression Study in Pulp and Seeds of Theobroma grandiflorum (Willd. ex Spreng.) Schum
Source: PLoS One. 2016 Aug 8;11(8):e0160646. doi: 10.1371/journal.pone.0160646 (PMC4976894; doi:10.1371/journal.pone.0160646)
Supplement: S2 Table — (DOCX) [file pone.0160646.s003.docx]

**S2 Table.** Amplicons characteristics

| Gene | Size (bp) | Tm (°C) | GC% | A | T | C | G | AT/GC |
| --- | --- | --- | --- | --- | --- | --- | --- | --- |
| ACP | 177 | 94.6 | 42.9 | 49 | 52 | 40 | 36 | 1.33 |
| ACT | 153 | 94 | 41.8 | 57 | 32 | 36 | 28 | 1.39 |
| GAPDH | 154 | 95 | 46.8 | 35 | 47 | 23 | 49 | 1.24 |
| MDH | 164 | 90 | 38.4 | 49 | 52 | 30 | 33 | 1.60 |
| TUB | 215 | 95 | 47.4 | 54 | 59 | 27 | 75 | 1.10 |
