# Supplementary material for: Selection of Reference Genes for Expression Study in Pulp and Seeds of Theobroma grandiflorum (Willd. ex Spreng.) Schum
Source: PLoS One. 2016 Aug 8;11(8):e0160646. doi: 10.1371/journal.pone.0160646 (PMC4976894; doi:10.1371/journal.pone.0160646)
Supplement: S3 Table — Maximum and minimum are indicated in bold. (*) indicates two maximum values for the considered gene. (DOCX) [file pone.0160646.s004.docx]

**S3 Table.** Ct mean of each reference candidate gene in the different tissues and stages analyzed. Maximum and minimum are indicated in bold. (*) indicated two maximum values for the considered gene.

|  | ACP | ACT | GAPDH | MDH | TUB |
| --- | --- | --- | --- | --- | --- |
| All stages and tissues | **24.0** | **21.3*** | **21.3** | **24.9** | 23.3 |
| Pulp (all stages) | 23.8 | 19.0 | 19.8 | 23.5 | 21.8 |
| Seeds (all stages) | **22.2** | **21.3*** | 20.3 | 24.2 | 22.4 |
| Young fruit (all tissues) | 23.1 | **18.7** | **19.8** | **23.1** | **20.7** |
| Maturing fruit (all tissues) | 22.8 | 21.0 | 20.0 | 24.6 | 22.2 |
| Mature fruit (all tissues) | 23.0 | 20.7 | 20.3 | 23.9 | **23.5** |
