# Supplementary material for: Selection of Reference Genes for Expression Study in Pulp and Seeds of Theobroma grandiflorum (Willd. ex Spreng.) Schum
Source: PLoS One. 2016 Aug 8;11(8):e0160646. doi: 10.1371/journal.pone.0160646 (PMC4976894; doi:10.1371/journal.pone.0160646)
Supplement: S4 Table — The asterisk indicates the lowest V value in each condition. Bold indicate the variation ≤ 0.15 (suggested cut-off). (DOCX) [file pone.0160646.s005.docx]

**S4 Table.** Estimation of the optimal number of reference genes required for accurate normalization based on pairwise variation (Vn/n+1) analysis. The asterisk indicates the lowest V value in each condition. Bold indicate the variation ≤ 0.15 (suggested cut-off).

|  | Pairwise variation | | | Optimal gene number^a^ |
| --- | --- | --- | --- | --- |
|  | V2/3 | V3/4 | V4/5 |  |
| All stages and tissues | 0.297 | 0.367 | 0.218* | At least 4 |
| Pulp (all stages) | 0.272 | **0.141*** | 0.299 | 3 |
| Seeds (all stages) | 0.526 | **0.142*** | 0.255 | 3 |
| Young fruit (all tissues) | 0.54 | 0.269 | **0.152*** | 4 |
| Maturing fruit (all tissues) | 0.689 | 0.458 | 0.361* | At least 4 |
| Mature fruit (all tissues) | 0.22 | **0.105** | **0.08*** | 3 |

^a^ required to pass the suggested cut-off value (0.15)
